# Supplementary material for: Candida utilis yeast as a functional protein source for Atlantic salmon (Salmo salar L.): Local intestinal tissue and plasma proteome responses
Source: PLoS One. 2019 Dec 30;14(12):e0218360. doi: 10.1371/journal.pone.0218360 (PMC6936787; doi:10.1371/journal.pone.0218360)
Supplement: S1 Table — (DOCX) [file pone.0218360.s001.docx]

**Supplementary Table 1. Chemical composition (g/kg) of inactivated dry *Candida utilis* biomass.**

| *Parameter* | *Candida utilis* |
| --- | --- |
| Dry matter | 920.5 |
| Crude protein | 391.0 |
| Crude fat | 21.4 |
| Gross energy (MJ/kg) | 18.4 |
| Ash | 67.0 |
|  |  |
| *Essential amino acids* |  |
| Arginine | 17.0 |
| Histidine | 6.5 |
| Isoleucine | 14.8 |
| Leucine | 22.9 |
| Lysine | 23.6 |
| Phenylalanine | 14.3 |
| Threonine | 17.7 |
| Valine | 17.4 |
| Methionine | 4.4 |
