# Supplementary material for: Candida utilis yeast as a functional protein source for Atlantic salmon (Salmo salar L.): Local intestinal tissue and plasma proteome responses
Source: PLoS One. 2019 Dec 30;14(12):e0218360. doi: 10.1371/journal.pone.0218360 (PMC6936787; doi:10.1371/journal.pone.0218360)
Supplement: S2 Table — (DOCX) [file pone.0218360.s002.docx]

Supplementary Table 2. Primer used in qPCR analyses.

| Protein name; Genbank accession | Gene name | Primer sequence | Amplicon size (bp)/  PCR efficiency | Reference |
| --- | --- | --- | --- | --- |
| Aquaporin-8ab; DW532464.1 | AQP8 | F: GTTGGCATAGTTCTCCTTTGATG  R: TTTCAACCCTCCCTTCACC | 148/1.96 | [1] |
| Catalase; XM_014131256.1 | CAT | F: CCAGATGTGGGCCGCTACAA  R: TCTGGCGCTCCTCCTCATTC | 94/2.03 | [2] |
| Superoxide dismutase 1; NM_001123587.1 | SOD | F: CCACGTCCATGCCTTTGG  R: TCAGCTGCTGCAGTCACGTT | 141/2.04 | [3] |
| Glyceraldehyde-3-phosphate dehydrogenase; BT050045.1 | GAPDH | F: AAGTGAAGCAGGAGGGTGGAA  R: CAGCCTCACCCCATTTGATG | 96/2.00 | [1] |
| Hypoxanthine phospho-ribosyltransferase 1; BT043501 | HPRTI | F: CCGCCTCAAGAGCTACTGTAAT  R: GTCTGGAACCTCAAACCCTATG | 238/2.02 | [1] |
| Annexin A1; CA060324 | ANXA | F: GTCAGAATCTTGGTCCTGGTTC  R: ACTGCCGTAGTGAAGTGTGCT | 98/1.91 | [1] |
| Glutathione S-transferase 3^a^; NM_001140755.1 | GSTA3 | F: AACGCCCAGAAATAGCCTCT  R: GACACGATTCATCCTCAGCA | 101/1.98 | [3] |

^a^ GSTA3 is referred to as GSTA4 in Sahlmann et al. (2013) however, after blasting against Atlantic salmon (taxid:8030) the resulting alignment were 100% with Salmo salar glutathione S-transferase alpha 3 (GSTA3) (NM_001140755.1).

1. Kortner T, Skugor S, Penn M, Mydland L, Djordjevic B, Hillestad M, et al. Dietary soyasaponin supplementation to pea protein concentrate reveals nutrigenomic interactions underlying enteropathy in Atlantic salmon (*Salmo salar*). BMC Veterinary Research. 2012;8(1):101. PubMed PMID: doi:10.1186/1746-6148-8-101.

2. Skugor S, Grisdale-Helland B, Refstie S, Afanasyev S, Vielma J, Krasnov A. Gene expression responses to restricted feeding and extracted soybean meal in Atlantic salmon (*Salmo salar* L.). Aquaculture Nutrition. 2011;17(5):505-17. doi: 10.1111/j.1365-2095.2010.00832.x.

3. Sahlmann C, Sutherland BJ, Kortner TM, Koop BF, Krogdahl A, Bakke AM. Early response of gene expression in the distal intestine of Atlantic salmon (Salmo salar L.) during the development of soybean meal induced enteritis. Fish Shellfish Immunol. 2013;34(2):599-609. doi: 10.1016/j.fsi.2012.11.031. PubMed PMID: 23246810.
