# Supplementary figures and images for: Candida utilis yeast as a functional protein source for Atlantic salmon (Salmo salar L.): Local intestinal tissue and plasma proteome responses
Source: PLoS One. 2019 Dec 30;14(12):e0218360. doi: 10.1371/journal.pone.0218360 (PMC6936787; doi:10.1371/journal.pone.0218360)

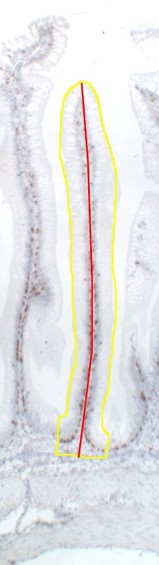

Supplement: S1 Fig — Red line indicates the measurement of fold height from the tip of the simple fold to the stratum compactum. The yellow line indicates the fold area including the simple fold and the lamina propria adjacent to the stratum compactum. (JPG) [file pone.0218360.s004.jpg]

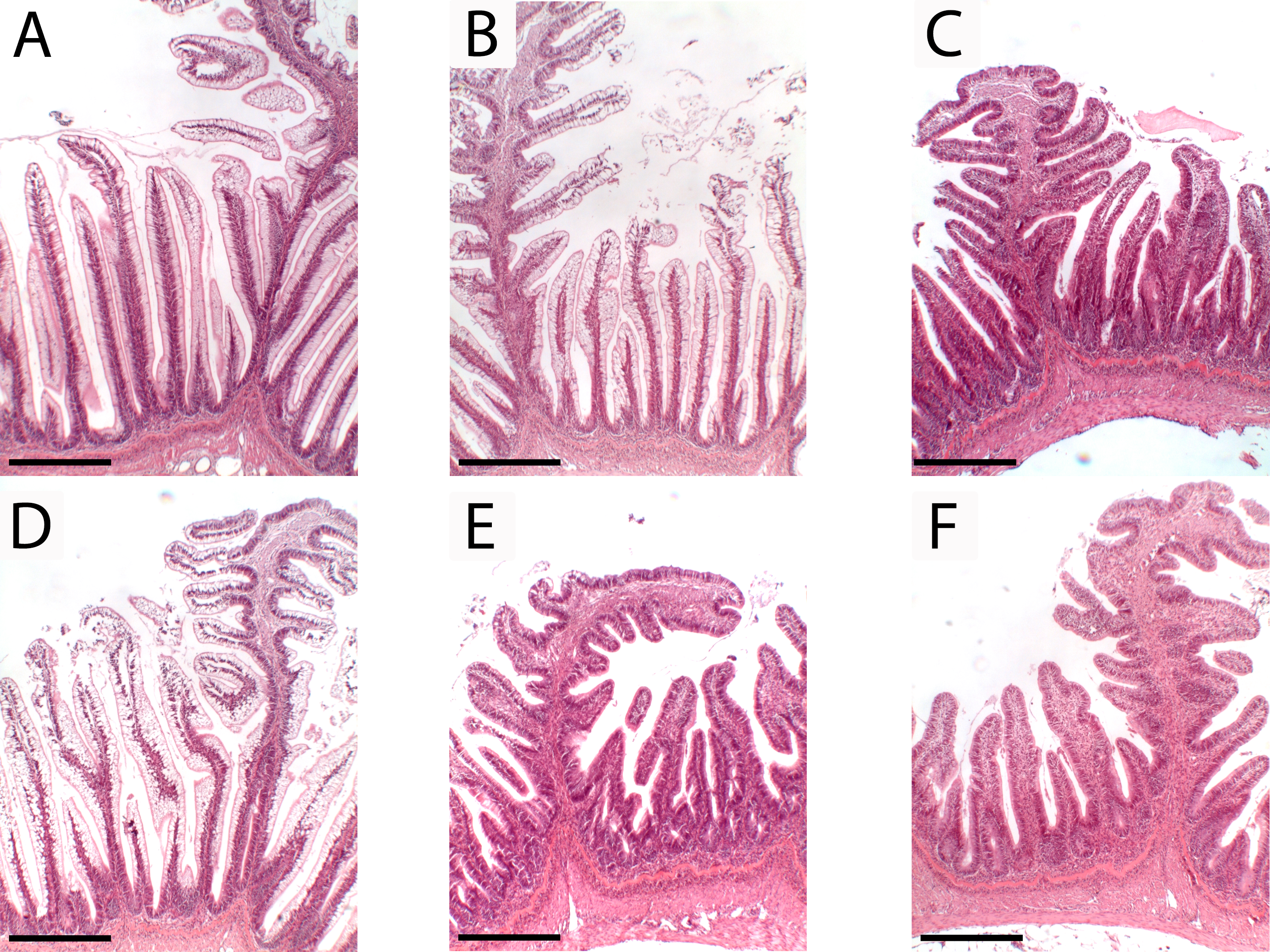

Supplement: S2 Fig — Representative histomorphological images from hematoxylin and eosin-stained sections of the distal intestine of Atlantic salmon fed control FM diet (A) and experimental diets (B-F). Normal morphology was seen in FM (A) and FM200CU (B) groups. Moderate changes associated with SBMIE was observed in the distal intestine of salmon fed SBM (C: SBM25CU, E: SBM200CU, F: SBM). Low inclusion of C. utilis to the SBM diet showed variation within the group ranging from individuals showing little changes (D: SBM25CU) to individuals with moderate changes in DI morphology (C: SBM25CU). All images are captured at 4x magnification with a scale bar (100μm). (TIF) [file pone.0218360.s005.tif]

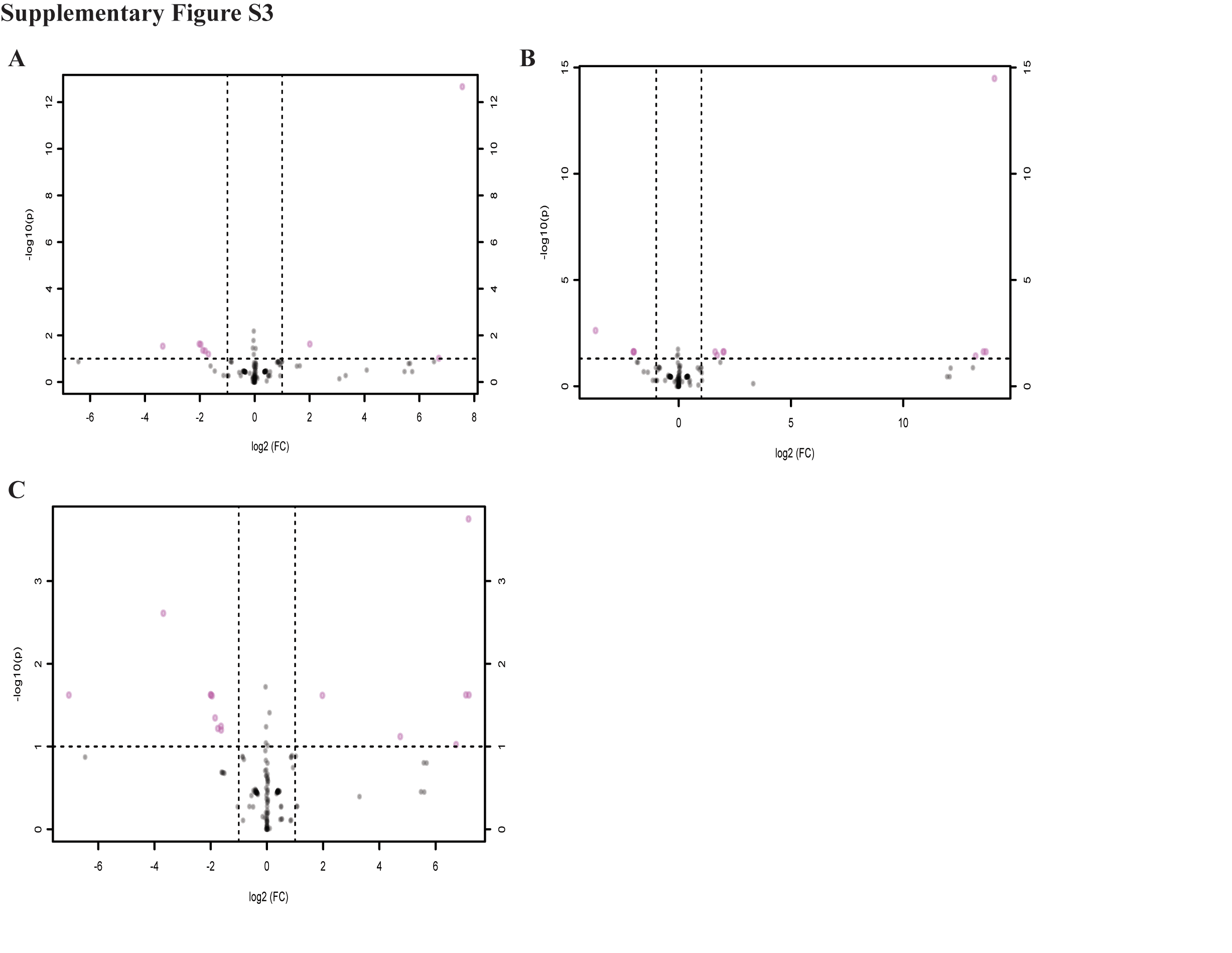

Supplement: S3 Fig — Volcano plots showing the relative expression of plasma proteins from fish fed SBM (A), SBM200CU (B) or FM200CU (C) compared to the control (FM). ANOVA plot with p-value threshold 0.05. (TIF) [file pone.0218360.s006.tif]
