# Supplementary material for: Candida utilis yeast as a functional protein source for Atlantic salmon (Salmo salar L.): Local intestinal tissue and plasma proteome responses
Source: PLoS One. 2019 Dec 30;14(12):e0218360. doi: 10.1371/journal.pone.0218360 (PMC6936787; doi:10.1371/journal.pone.0218360)
Supplement: Attachment — Submitted filename: Response to Reviewers.docx [file pone.0218360.s008.docx]

6. Review Comments to the Author

Please use the space provided to explain your answers to the questions above. You may also include additional comments for the author, including concerns about dual publication, research ethics, or publication ethics. (Please upload your review as an attachment if it exceeds 20,000 characters)

Reviewer #1: (No Response)

Reviewer #2: I have now been through this revised version of the manuscript. I am generally happy with all the authors responses to my original review. So I am happy to hereby recommend the manuscript for publication.

But I do have a few minor comments to help with some final improvements:

1)

Regarding the Y-axes labels on figure 2, it may be more appropriate to label them "Change" rather than "Scale" that seems not very informative.

The label on the Y-axis has been changed from "scale" to "change" according to the reviewer’s request.

2)

I suggest that the authors either delete or re-write the very kast sentence of the discussion:

"Further research is needed to evaluate the impact of yeast strain and the fermentation and/or down-stream processing conditions of the yeast on functional properties in relation to gastro intestinal health and systemic responses."

As is, this is not very informative. Consider adding some more concrete suggestions as to how such "Further research" can be designed to better help move this entire research field and add more values to readers of the paper.

The last sentence has been deleted as the discussion has been changed.

Reviewer #3: The authors addressed a number of concerns raised by the reviewers and the manuscript has been significantly improved. Unfortunately, this reviewer still has significant concerns about the analysis of the proteomics data and the conclusions that are drawn from this data.

In the original manuscript, it was not made clear that the authors were utilizing spectral counting to obtain quantitative information from the proteomics data. This should have been stated in the manuscript, and needs to be added. The spectral counting method has been phased out in the field due to its low reproducibility and accuracy. When using a high resolution, accurate mass instrument such as the Q Exactive that was used here, MS1 intensity-based quantitation provides far superior quantitative values than does spectral counting. The reasons for this are well described in the literature, but are primarily the result of the somewhat stochastic nature of the data collection when data-dependent acquisition strategies are employed (as was done in this study). The fact that an MS2 mass tolerance was used for searching that was more than an order of magnitude higher than it should have been (0.8 Da rather than 0.02 Da) further reduces the quantitative accuracy of the spectral counting strategy used here.

For the reasons described above, the quantitative values presented should be treated with caution. The proteins shown to be differentially expressed in the volcano plots (Fig. 7) are likely valid, as this analysis incorporates both the calculated fold change, as well as a p-value, which incorporates other variables such as inter-sample variability. Unfortunately, many of the proteins that the authors focus on in the discussion (such as HMP, LYME, and HIIN) were not shown to be differentially expressed by this analysis, they simply didn't correlate well to the control. This alone is not adequate evidence of a change in protein abundance.

With these concerns in mind, this reviewer suggests the authors choose one of 3 options:

1. Reanalyze the data using MS1 intensity-based quantitation (as can be completed in MaxQuant or other software packages such as Scaffold Q+, Proteome Discoverer, etc.).

2. Focus the discussion only on proteins that showed differential abundance in the volcano plot analyses (Fig. 7)

3. Remove the proteomics data entirely and focusing on the gene expression, morphological, and other data presented in the manuscript.

Any of the above approaches would be valid and would make the publication suitable for publication in the opinion of this reviewer.

We are grateful for the specificity and professional comments of reviewer 3. We decided to take option number 1, which means at we reanalyzed the entire dataset using MaxQuant and Perseus. The LFQ intensities were exported to an excel sheet. The log-transformed LFQ values were analyzed using the pipeline described in material and methods. We created volcano plots, comparing the protein pattern of each dietary treatment with the control diet. This comparison resulted in 9, 12 and 10 significantly expressed protein when the FM was compared with SBM, SBM200CU and FM200CU, respectively. Although option 1 was more complicated, we are very grateful for the comments, which helped us to increase the reliability of our results.

Specific Comments:

L262: The date the database was downloaded should also be included.

We used the latest salmon proteome available and the date is included in the manuscript

L403: Don't say 'expressed' here. The proteins were likely expressed in the samples, they just couldn't be detected by the mass spectrometer.

The sentence was changed as marked in the manuscript.

Figure 6: The Spearman's correlations done here do not provide sufficient evidence to say a protein is changing abundance. Panel C should be removed from the manuscript as well as all discussion of the results presented in this panel.

In accordance with the reviewer’s advice, Panel C in Figure 6 has been removed from the manuscript and the discussion revised accordingly.
