# Supplementary material for: Candida utilis yeast as a functional protein source for Atlantic salmon (Salmo salar L.): Local intestinal tissue and plasma proteome responses
Source: PLoS One. 2019 Dec 30;14(12):e0218360. doi: 10.1371/journal.pone.0218360 (PMC6936787; doi:10.1371/journal.pone.0218360)
Supplement: Attachment — Submitted filename: Response to Reviewers.docx [file pone.0218360.s009.docx]

Additional Editor Comments (if provided):

- Please address the possible data discrepancies between table S3 and table 2 referred to by Reviewer 3;

- Make sure that proteins that were not found in a replicate are not given a value of zero but treated as missing in your analysis (or clearly state that you did not use 0 in your analysis)

- Make the minor adjustments to text suggested by Reviewer 3.


Reviewers' comments:

Reviewer's Responses to Questions

**Comments to the Author**

1. If the authors have adequately addressed your comments raised in a previous round of review and you feel that this manuscript is now acceptable for publication, you may indicate that here to bypass the “Comments to the Author” section, enter your conflict of interest statement in the “Confidential to Editor” section, and submit your "Accept" recommendation.

Reviewer #3: (No Response)
 
2. Is the manuscript technically sound, and do the data support the conclusions?

The manuscript must describe a technically sound piece of scientific research with data that supports the conclusions. Experiments must have been conducted rigorously, with appropriate controls, replication, and sample sizes. The conclusions must be drawn appropriately based on the data presented.

Reviewer #3: Yes

3. Has the statistical analysis been performed appropriately and rigorously?

Reviewer #3: Yes

4. Have the authors made all data underlying the findings in their manuscript fully available?

The [PLOS Data policy](http://www.plosone.org/static/policies.action#sharing) requires authors to make all data underlying the findings described in their manuscript fully available without restriction, with rare exception (please refer to the Data Availability Statement in the manuscript PDF file). The data should be provided as part of the manuscript or its supporting information, or deposited to a public repository. For example, in addition to summary statistics, the data points behind means, medians and variance measures should be available. If there are restrictions on publicly sharing data—e.g. participant privacy or use of data from a third party—those must be specified.

Reviewer #3: Yes

5. Is the manuscript presented in an intelligible fashion and written in standard English?

PLOS ONE does not copyedit accepted manuscripts, so the language in submitted articles must be clear, correct, and unambiguous. Any typographical or grammatical errors should be corrected at revision, so please note any specific errors here.

Reviewer #3: Yes

6. Review Comments to the Author

Please use the space provided to explain your answers to the questions above. You may also include additional comments for the author, including concerns about dual publication, research ethics, or publication ethics. (Please upload your review as an attachment if it exceeds 20,000 characters)

Reviewer #3: General Impressions:

The authors have clearly addressed the concerns raised in the previous review, and the reanalysis of the proteomics data has dramatically improved the quality of the manuscript. I greatly appreciate the authors hard work and dedication to providing the highest quality data possible. I have noticed one discrepancy in the data analysis that should be addressed in the final version of the manuscript (described below), but I feel that the manuscript is otherwise ready for publication. I’ve also included a few very minor comments that I feel will improve the readability of the publication.

Major Comments:

The authors state that only protein that were identified in at least two of the four replicates were used for quantitative analysis. However, when looking at Table S3 and comparing that to the results presented in Table 2, there seems to be some discrepancies. For example, C0HAL2 (elongation factor 1-alpha) is reported as having a very significant fold-change between the FM (D1) and SBM (D2) in Table 2, yet no quantitative values for this protein are reported in the D2 group in Table S3. Why is this? Please ensure that your filtering is working as intended.

We follow the criteria “at least two peptides in at least half of the replicates” per group. It means to asseverate that a protein is present in one or all the groups, should be at least in two samples. As an example, protein C0HAL2 is detected in all replicated of diet 1, in one of the replicates on diet 6 and in three out of the four replicates on diet 7. It was not detected on diet 2, therefore the significant difference between diet 1 and diet 2. From the total 158 proteins, 126 proteins were present in all dietary treatment, however the other 32 proteins were present in either one, two or three of the treatment, not in all, as stated in the manuscript.

Additionally, proteins that were not identified in a replicate should not be assigned a quantitative value of 0. Instead, these values should be treated as missing (null), or missing value imputation should be used to provide baseline quantitative values. It’s difficult to determine how such values were treated in the analysis, and that should be stated outright.

The function missing value imputation was used for the quantitative analysis. The imputed value is shown in Table S3 and it is mentioned in the manuscript as follow:

“Protein raw data were transferred to log normalization; missing value imputation was used to replace the not identified proteins on the quantitative analysis and then performed on autoscaled data (mean-centered and divided by the standard deviation of each variable) [29].”

As an aside for future experiments, use of the ‘match between runs’ feature in MaxQuant should dramatically decrease the number of missing values present in the dataset.

We are very grateful for the suggestions, we have been learning a lot in this process so we are confident that in future experiments we will take into practice the knowledge acquired.

Minor Comments:

Line 241: It seems as if part of this sentence was accidentally removed as there is no mention of the addition of trypsin.

Yes, it was an editing mistake. The sentence has been rewritten as follow:

“Subsequently, the proteins were digested with 10 μg trypsin (Promega, sequencing grade) overnight at 37°C. The digestion was stopped by adding 5 μL 50% formic acid and the generated peptides were purified using a ZipTip C18 (Millipore, Billerica, MA, USA) according to the manufacturer’s instructions, and dried using a Speed Vac concentrator (Concentrator Plus, Eppendorf, Hamburg, Germany)”

Line 243: Another sentence or two very briefly describing the column and MS parameters that were used would be useful here.

The information required was added into the text

“The tryptic peptides were dissolved in 10 µL 0.1% formic acid/2% acetonitrile and 5 µL analyzed using an Ultimate 3000 RSLCnano-UHPLC system connected to a Q Exactive mass spectrometer (Thermo Fisher Scientiﬁc, Bremen, Germany) equipped with a nanoelectrospray ion source. For liquid chromatography separation, an Acclaim PepMap 100 column (C18, 2 µm beads, 100 Å, 75 μm inner diameter, 50 cm length) (Dionex, Sunnyvale CA, USA) was used. The mass spectrometer was operated in the data-dependent mode to automatically switch between MS and MS/MS acquisition. Survey full scan MS spectra (from m/z 400 to 2,000) were acquired with the resolution R = 70,000 at m/z 200, after accumulation to a target of 1e5. The maximum allowed ion accumulation times were 60 ms”

Line 261: Was a fragment mass tolerance of 0.8 used in the reprocessed data as well or was this a carryover from the previous version of the manuscript? As mentioned previously, a much tighter tolerance (~0.02 Da) would be more appropriate.

This corresponded to the previous analysis, the new analysis was performed using:

“The fragment mass tolerance for the MS1 was 6 ppm and the fragment mass tolerance for the MS2 was 20 ppm”.

This information was added into the text

Lines 276-277: Saying that the average weights increased does not mean that every fish gained weight. Consider rephrasing.

The sentence was rephrased as follow:

“The average initial weight was 526 g and the average final 277 weight was 667 g on day 37, considering that the weight was measured as bulk, this indicates that in general fish gained weight during the experimental period”

Table 2: It would be nice if you included the p-value for each protein here as well.

We have included the p-value in Table 2 as required by Reviewer 3.

Figure 6: As a note, the ‘presence’ of a protein in one sample and not in another is much more likely a result of the stochastic nature of the MS data collection rather than a protein actually being present in the sample or not. Therefore, comparisons such as the one shown here are often not very informative.

 We are grateful for the comments and it will be considered in our future experiments.

7. PLOS authors have the option to publish the peer review history of their article ([what does this mean?](https://journals.plos.org/plosone/s/editorial-and-peer-review-process#loc-peer-review-history)). If published, this will include your full peer review and any attached files.


If you choose “no”, your identity will remain anonymous but your review may still be made public.


**Do you want your identity to be public for this peer review?** For information about this choice, including consent withdrawal, please see our [Privacy Policy](https://www.plos.org/privacy-policy).

Reviewer #3: No
